# Sport advice given by Dutch orthopaedic surgeons to patients after a total hip arthroplasty or total knee arthroplasty

**DOI:** 10.1371/journal.pone.0202494

**Published:** 2018-08-30

**Authors:** Sieger Bertus Meester, Robert Wagenmakers, Inge van den Akker-Scheek, Martin Stevens

**Affiliations:** 1 Department of Orthopaedics, University of Groningen, University Medical Center Groningen, Groningen, The Netherlands; 2 Department of Orthopaedics, Amphia Hospital Breda, Breda, The Netherlands; Consorci Parc de Salut MAR de Barcelona, SPAIN

## Abstract

**Background:**

The advice given to patients in the Netherlands regarding sport activities after total hip arthroplasty or total knee arthroplasty (THA/TKA) is currently based on the opinion of the individual orthopaedic surgeon.

**Aim:**

To give an overview of the sport advice given by Dutch orthopaedic surgeons and to examine whether surgeons are familiar with the Dutch health-enhancing physical activity (PA) recommendations (NNGB).

**Methods:**

472 surgeons were selected to fill in a questionnaire regarding 40 sport activities for four patient age groups (in years): 1) THA<65, 2) THA>65, 3) TKA<65 4) TKA>65. Surgeons were also asked if they discuss the role of PA postoperatively and about their knowledge and application of the NNGB.

**Results:**

There was consensus on 29 sport activities for the THA<65 group and 30 activities for the THA>65 group. In the TKA<65 group there was consensus for 33 sports activities and in the TKA>65 group for 32 activities. Amongst orthopaedic surgeons performing THAs and TKAs, respectively 77% and 79% discussed the role of PA postoperatively with their patients, and a total of 34% and 41% were familiar with the NNGB, with 33% and 34% of them giving NNGB-based advice.

**Conclusion:**

Results can be used to recommend sport activities after THA/TKA. Although the majority of orthopaedic surgeons discuss the role of PA postoperatively with their patients, familiarity with health-enhancing PA recommendations is lacking.

## Introduction

Total Hip Arthroplasty (THA) and Total Knee Arthroplasty (TKA) are cost-effective, pain-relieving treatments for end-stage osteoarthritis, and improve the ability to stay physically active[1]. After THA or TKA it is of the utmost importance that surgeons advice patients to adopt a physically active lifestyle, not only from a general health perspective but also because physical activity (PA) benefits the prosthesis[2].

There is overwhelming evidence stating that PA prevents several chronic diseases and decreases mortality[2, 3]. PA also improves overall health and fitness, which among other things can improve the ability to keep up activities of daily living (ADL) independently while aging[2, 4–6]. THA and TKA patients can benefit even more from PA. There are indications that PA results in lower fall risk, increased bone density, improved prosthetic fixation and reduced risk of prosthetic loosening[7]. On the other hand, excessive or inappropriate PA can negatively influence prosthetic wear and loosening, affecting the longevity of the hip or knee prosthesis[8–10].

Strikingly, by contrast doctors do not pay much attention to discussing the role of PA with their patients. For example, less than a quarter of medical students and junior doctors ask patients about their PA levels when taking their medical history, whereas more than 90% always ask about tobacco and alcohol use[11].

It is essential that THA/TKA patients be counselled correctly both before and after surgery about the benefits of a physically activity lifestyle and taking up or returning to sport activities after surgery. Moreover, for those who were physically active before surgery, returning to sport activities is often an important expectation[12, 13]. Surgeons are thus increasingly asked what kind of sport activity is advised after THA/TKA[13]. Two American studies, one Danish and one British study presented a consensus-based guideline for sport activities. One of the American studies looked at activities after THA, stating that low-impact sport activities such as swimming, biking and walking were allowed[14]. Their British colleagues presented a similar guideline for THA patients whereby surgeons also allowed patients to return to low-impact activities[15]. American research on sport activities after both THA and TKA concluded that surgeons recommend walking, swimming and biking on level surfaces, and higher-impact activities such as contact sports are generally discouraged[16]. The Danish study also looked at the recommendations given after both THA and TKA, reporting fairly similar results yet concluding that the recommendations for TKA are less liberal than those for THA patients[17].

A problem arises when these guidelines are applied to the Dutch situation, because to a certain extent sports activities are culturally determined. Moreover, there are no Dutch evidence-based clinical practice guidelines. The advice currently given is thus solely based on the opinion and experience of the individual treating orthopaedic surgeon. Recognition of the importance of PA has led to international recommendations for health-enhancing physical activity. The Dutch health-enhancing PA recommendation (NNGB) is based on these international recommendations[18, 19]. The NNGB states that 30 minutes of moderate-intensity exercise 5 days a week will yield health benefits. Meeting the health norm however does not necessarily mean a person is physically fit. Hence the fitnorm was introduced, stating that 20 minutes of high-intensity sport/exercise 3 days a week will not only yield the same benefits as the health norm, but will also enable persons to remain fit. Meeting the fitnorm can eventually improve the independence of older adults. For the above reasons, it is relevant to make an inventory of the advice given in the Netherlands. Aim of the study is therefore to inventory and provide an overview of the sport advice given by orthopaedic surgeons after THA and TKA. Secondary aim is to examine to what extent surgeons are familiar with the NNGB.

## Method

### Population

Orthopaedic surgeons registered by the Netherlands Orthopaedic Association (NOV) were screened for selection. Inclusion criteria were surgeons who are (1) registered in the Dutch databank for health professionals; and (2) currently working in the Netherlands. Exclusion criteria were (1) either a non-working e-mail address or an unlisted e-mail address in the NOV database; and (2) surgeons who did not perform THA or TKA surgeries.

### Procedure

From September 2009 to February 2010, surgeons were invited by email to fill in an online questionnaire. A reminder e-mail was sent after two weeks. The study was conducted in accordance with the Dutch law (WMO) and regulations of the local medical ethical committee. As no patients were involved in the study there was no need for approval from the Medical Ethics Committee of University Medical Center Groningen (UMCG). Data were processed anonymously in a secured database on the server of UMCG.

### Questionnaire

A division was made between surgeons that performed either THA or TKA or both. Surgeons who performed both procedures had to fill in a questionnaire for each surgery. A distinction was made between people under and over age 65, so surgeons had to fill in a questionnaire for each age group. The distinction between the two age groups is based on changes in motor fitness between younger and older adults and their consequences for sports participation[20]. After the introductory questions, surgeons were asked how often they performed THA/TKA. The remaining design of the questionnaire was based on previous American research listing 37 sports activities[14]. The present study made an adaptation to the Dutch situation by inserting the most practiced sport activities for older adults[18]. The final questionnaire consisted of 40 sport activities (see Table 1). For each sport activity, surgeons could indicate if 1) it was allowed, 2) it was allowed when the patient had experience with the activity, 3) it was discouraged, or 4) they had no opinion[14]. ‘Allowed with experience’ was defined as the basic technique of the sport activity being correctly performed. Finally, questions on knowledge about and application of the NNGB were asked.

**Table 1:** Sport advice after THA.

Sport	THA<65 years	THA>65 years
Aerobics	Allowed with experience	Allowed with experience
Aqua fitness	Allowed	Allowed
Badminton	No advice	No advice
Basketball	Discouraged	Discouraged
Bicycling	Allowed	Allowed
Canoeing	Allowed with experience	Allowed with experience
Cross-country skiing	Allowed with experience	Allowed with experience
Cross-walking	No advice	No advice
Cycling	Allowed with experience	Allowed with experience
Dancing	Allowed	Allowed
Fitness	Allowed	Allowed
Football—field	Discouraged	Discouraged
Football–hall	Discouraged	Discouraged
Fysiofitness	Allowed	Allowed
Golf	Allowed	Allowed
Gymnastics	No advice	No advice
Handball	Discouraged	Discouraged
Hockey	No advice	Discouraged
Ice skating	Allowed with experience	Allowed with experience
Jeu de boules (game of bowls)	Allowed	Allowed
Koersbal (bowls)	No advice	No advice
Korfbal (korf ball)	Discouraged	Discouraged
Martial arts	Discouraged	Discouraged
Nordic walking	Allowed	Allowed
Horseback riding	Allowed with experience	Allowed with experience
Rowing	Allowed with experience	Allowed with experience
Running	No advice	No advice
Running on a treadmill	No advice	No advice
Sailing	Allowed with experience	Allowed with experience
Skiing	Allowed with experience	No advice
Snowboarding	No advice	Discouraged
Squash	No advice	No advice
Surfing	No advice	No advice
Swimming	Allowed	Allowed
Table tennis	Allowed with experience	Allowed with experience
Tennis—singles	Allowed with experience	No advice
Tennis—doubles	Allowed with experience	Allowed with experience
Volleyball	No advice	Discouraged
Walking	Allowed	Allowed
Yoga/Tai-chi	Allowed with experience	Allowed with experience

Notes: Table 1 presents the results whether the sport was allowed, allowed with experience, not allowed or no advice was given after a THA for the two age-groups.

### Data analysis

Analyses were performed using SPSS (IBM SPSS, version 16.0, Chicago). Questionnaires were excluded if they were completed by less than 10%. If a respondent had completed a questionnaire twice, the second or less completed questionnaire was excluded. Proportions were tested using the Z-score. Based on the number of respondents per sport activity, a power analysis for a 1-sample proportion test was performed to determine the percentage needed to reach statistical significance (p < 0.05) in one of the four categories: (1) allowed sports activities, 2) sports activities allowed with experience, 3) discouraged sports activities, or 4) no advice. For each type of activity the response percentage in every category was compared to the percentage that was needed to reach statistical significance with a Z-test. If statistical significance was achieved for one of the activities in one of four categories, then consensus for that category was stated. If an activity did not reach significance in one of the four categories, then the categories ‘allowed’ and ‘allowed with experience’ were combined. Next it was calculated whether the combined categories achieved the required percentage in order to reach significance. If significance was reached, consensus in the ‘allowed with experience’ category was stated for that activity. ‘No advice’ was stated when either 1) the (combined) category did not reach significance, or 2) the ‘no opinion’ category reached significance. Finally, it was determined whether there was a difference in consensus per activity among the different age groups.

## Results

**Fig 1:**
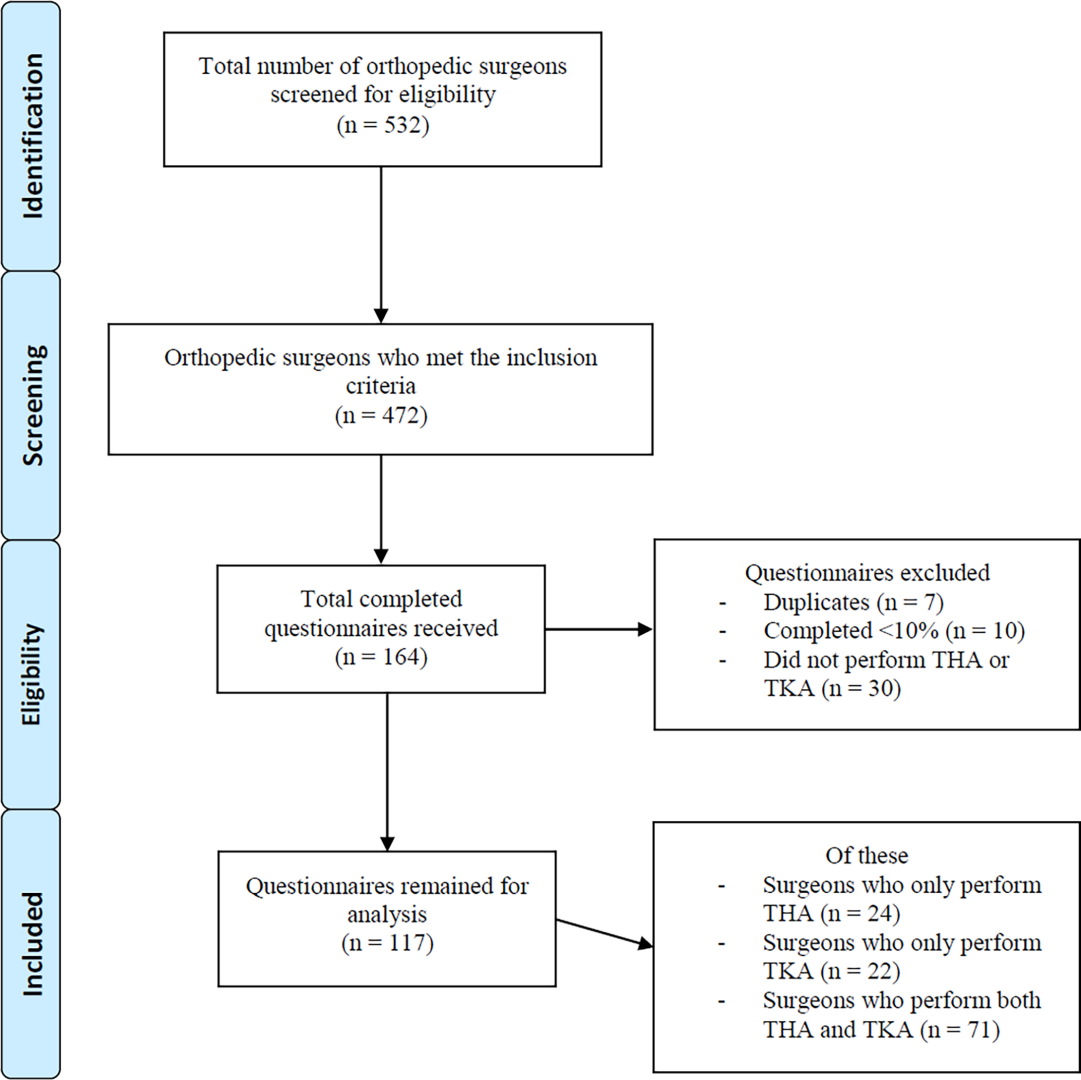
Flow diagram of inclusion of participants.

Results of the orthopaedic surgeons’ selection procedure are presented in Fig 1. The total number of orthopaedic surgeons in the NOV (n = 532) file were screened for eligibility; 472 surgeons met the inclusion criteria and were approached to complete the questionnaire. Finally, 117 respondents remained for analysis who were responsible for 164 (34.7%) completed questionnaires (THA, TKA, and THA & TKA)

### THA under age 65

Ten sport activities achieved consensus for the ‘allowed’ category; on average, 91% (82.8%–96.8%) of the surgeons gave the advice ‘allowed’. Thirteen activities achieved consensus for the ‘allowed with experience’ category; on average, 85% (69.9%–96.8%) gave the advice ‘allowed’ or ‘allowed with experience’. Finally, six activities achieved consensus for the ‘discourage’ category; on average, 80% (69.9%–88.0%) gave the advice ‘discourage’. Eleven activities did not reach consensus (see Table 1).

### THA over age 65

Ten sport activities achieved consensus for the ‘allowed’ category; on average, 91% (80.6%–95.7%) of the surgeons gave the advice ‘allowed’. Eleven activities achieved consensus for the ‘allowed with experience’ category; on average, 83.3% (74.2%–94.7%) gave the advice ‘allowed’ or ‘allowed with experience’. Finally, nine activities achieved consensus for the ‘discourage’ category; on average, 81% (71.0%–92.5%) gave the advice ‘discourage’. Ten activities did not reach consensus (see Table 1).

### TKA under age 65

Eleven sport activities achieved consensus for the ‘allowed’ category; on average, 90% (70.3%–96.7%) of the surgeons gave the advice ‘allowed’. Eleven activities achieved consensus for the ‘allowed with experience’ category; on average, 87.2% (68.9%–98.9%) gave the advice ‘allowed’ or ‘allowed with experience’. Finally, eleven activities achieved consensus for the ‘discourage’ category; on average, 77.8% (60.7%–92.3%) gave the advice ‘discourage’. Seven activities did not reach consensus (see Table 2).

**Table 2:** Sport advice after TKA.

Sport	TKA<65 years	TKA>65 years
Aerobics	Allowed with experience	Allowed with experience
Aqua fitness	Allowed	Allowed
Badminton	No advice	No advice
Basketball	Discouraged	Discouraged
Bicycling	Allowed	Allowed
Canoeing	Allowed with experience	Allowed with experience
Cross-country skiing	Allowed with experience	Allowed with experience
Cross-walking	No advice	Allowed with experience
Cycling	Allowed with experience	Allowed with experience
Dancing	Allowed	Allowed
Fitness	Allowed	Allowed
Football—field	Discouraged	Discouraged
Football–hall	Discouraged	Discouraged
Fysiofitness	Allowed	Allowed
Golf	Allowed	Allowed
Gymnastics	No advice	No advice
Handball	Discouraged	Discouraged
Hockey	Discouraged	Discouraged
Ice skating	Allowed with experience	Allowed with experience
Jeu de boules (game of bowls)	Allowed	Allowed
Koersbal (bowls)	No advice	No advice
Korfbal (korf ball)	Discouraged	Discouraged
Martial arts	Discouraged	Discouraged
Nordic walking	Allowed	Allowed
Horseback riding	Allowed with experience	Allowed with experience
Rowing	Allowed	Allowed with experience
Running	Discouraged	Discouraged
Running on a treadmill	Discouraged	No advice
Sailing	Allowed with experience	Allowed with experience
Skiing	No advice	No advice
Snowboarding	Discouraged	Discouraged
Squash	No advice	No advice
Surfing	Allowed with experience	No advice
Swimming	Allowed	Allowed
Table tennis	Allowed with experience	Allowed with experience
Tennis—singles	No advice	No advice
Tennis—doubles	Allowed with experience	Allowed with experience
Volleyball	Discouraged	Discouraged
Walking	Allowed	Allowed
Yoga/Tai-chi	Allowed with experience	Allowed with experience

Notes: Table 2 presents the results whether the sport was allowed, allowed with experience, not allowed or no advice was given after a TKA for the two age-groups.

### TKA over age 65

Ten sport activities achieved consensus for the ‘allowed’ category; on average, 90% (78.9%–96.7%) of the surgeons gave the advice ‘allowed’. Twelve activities achieved consensus for the ‘allowed with experience’ category; on average, 83.2% (71.5%–95.6%) gave the advice ‘allowed’ or ‘allowed with experience’. Finally, ten activities achieved consensus for the ‘discourage’ category; on average, 83% (74.4%–94.5%) gave the advice ‘discourage’. Eight activities did not reach consensus (see Table 2).

### Results for postoperative sport advice and NNGB

A total of 184 questionnaires were filled in (93 for THA, 91 for TKA). Seventy-seven percent of orthopaedic surgeons discuss sport activities after THA, 76% after TKA. Thirty-nine percent of surgeons who perform THAs are familiar with the NNGB and 33% advise patients to meet its recommendations. Forty-one percent of surgeons who perform TKAs are familiar with the NNGB and 34% advise patients to meet its recommendations (see Table 3).

**Table 3:** NNGB and sport activities after THA and TKA.

	THA			TKA		
	N	Yes (%)	No (%)	N	Yes (%)	No (%)
Mentions sport activities postoperatively to patient	93	72 (77.4)	21 (22.6)	91	66 (75.8)	22 (24.2)
Is familiar with the NNGB	93	36 (38.7)	57 (61.3)	91	37 (40.7)	54 (59.3)
Advises patient to meet the NNGB norm	93	31 (33.3)	62 (62.7)	91	31 (34.1)	60 (65.9)

Notes: Table 3 presents the results whether the orthopaedic surgeons mention sport activities after a surgery, if they were familiar with the NNGB and if they advise patients to meet the norm.

## Discussion

To our knowledge, this is the first study that aimed to inventory the advice given by Dutch orthopaedic surgeons on participation in sport activities after THA or TKA. Previous American, British and Danish research already presented consensus-based guidelines[14, 15, 17]. The present study found the highest number of activities that reached consensus in the TKA<65 category and the lowest in the THA<65 age category. Less than half of the surgeons reported being familiar with the NNGB, and only one-third of surgeons discuss the recommendations with their patients.

The Dutch results are in line with studies done in other countries. Recent British research reporting that all UK surgeons allowed patients to return to low-impact activities after THA[15]. In addition, a recent (2016) systematic review concluded that 94% of all surgeons allowed patients to return to low-impact sport activities after TKA[19]. Most ball sports, except for doubles tennis and table tennis, are generally not recommended after THA or TKA. These results are in line with those of Klein et al.[14]. For handball, basketball and volleyball the high shock load and frequent jumping and landing could be the explanatory variables for the high percentage in the ‘discouraged’ category. The high percentages in the ‘discouraged’ category for hockey, basketball, indoor soccer, outdoor soccer and snowboarding could be attributed to the high twisting forces on the joints as well as the large lateral and rearward forces on the joints that these activities entail[21]. Martial arts were discouraged across the entire board. This is in line with British research and the conclusion of the review of Vogel et al., who stated that patients should not participate in contact sports after THA or TKA[15, 22]. An American study also concluded that for both THA and TKA high-impact sports are discouraged and low-impact sports are allowed[16]. Other American research suggests that high-impact sports such as football, martial arts and tennis increase the wear rate and negatively affect implant survivorship after THA[23].

Whereas the recommendations of Dutch surgeons after a TKA are fairly similar to previous research, there seems to be a trend towards a more conservative recommendation after THA compared to American colleagues[16]. For example, in contrast to the present study the American survey found consensus in the ‘allowed’ category for cross-walking and running on a treadmill after THA[14]. Also for doubles tennis and aerobics after THA there was consensus among American surgeons that these belong to the ‘allowed’ category, while consensus in the ‘allowed with experience’ category among Dutch surgeons is a more conservative indication[14]. Danish surgeons allowed even more activities– 87% of athletic activities for both THA and TKA, 55% of them allowing for high-impact activities after THA compared to 21% in the American guideline[17]. Recommendations for TKA patients were however fairly similar[17]. These results are in line with American research stating that surgeons are more liberal in their recommendations after THA compared to TKA[16].

In all groups, except for TKA>65, a high percentage in the ‘no opinion’ category was found for cross-walking. As cross-walking has a low impact on the joint and because a general consensus was found in the category of other low-impact activities, Dutch surgeons are likely to be unfamiliar with this activity. This also applies to ‘bowls’, which also had a high percentage in the ‘no opinion’ category for all groups. However, for ‘jeu des boules’ (game of bowls) there is a strong consensus in the ‘allowed’ category for all groups. Given the similarity of bowls and jeu des boules and the differences in outcomes, it is likely that not every surgeon is familiar with bowls.

With respect to the NNGB, it is striking that less than half of Dutch surgeons who perform THA or TKA (39% and 41%, respectively) indicated being familiar with it. An even lower percentage (33% for THA and 34% for TKA) advises patients to actually meet the NNGB recommendations, yet more than three-quarters of the surgeons discuss sport activities after surgery with their patients. Hence not meeting the NNGB recommendations could be a matter of unfamiliarity with the norm. This finding seems to be in line with British research which concluded that doctors do not pay much attention to discussing the role of PA with their patients. Contrary to tobacco use and alcohol consumption, doctors tend to under-prioritise physical inactivity as a mortality risk factor, which may reflect a lack of adequate teaching and emphasis on physical activity in medical schools[8]. Nonetheless, advising patients to meet the norm is of the utmost importance, as regular PA has been indicated to improve overall health and fitness[2, 3]. To that end, a suitable sports activity, walking or cycling when commuting, or even taking the stairs can be advised. However, based on previous research it can be concluded that although patients increased their PA level postoperatively compared to preoperatively, most patients do not meet health-enhancing physical activity recommendations like the NNGB and are less active compared to healthy peers[24–27]. In addition to these overall health and fitness benefits, regular PA also has been indicated to increase bone density, improve prostatic fixation, reduce the risk of prosthetic loosening and prevent falls[28–30]. For those with a prosthesis, falls can result in periprosthetic fracture, implant loosing and/or dislocation of hip prosthesis[26].

The present study has several limitations. The first concerns the low response rate, although the results are in line with similar research[14]. For that reason it can be suggested that the results may be considered representative. Secondly, although the data dates from 2010, the results are in line with recent research and thus can still be applied to give advice[15, 17]. Thirdly, cultural differences within the Netherlands with respect to the sports activities included were not considered. These are however expected to play a minor role, as the selection of activities was based on a representative survey[7, 20]. Fourth, the focus of the study lay primarily on the advice given by orthopaedic surgeons for sports activities after a THA or TKA. This means that we did not primarily focus on the potentially beneficial effect of other physical activities, such as walking or cycling when commuting. These activities were nonetheless taken into account with respect to meeting NNGB recommendations. Finally, surgeons were not asked whether they participate in sport activities themselves. It can be expected that surgeons who participate in sport activities are more inclined to allow sport activities than surgeons who do not.

## Conclusion

Results of this survey can be used to recommend sport activities after THA or TKA in the Netherlands as part of an overall active lifestyle. Surgeons allowed the most sports activities after TKA, and the least after THA. Even though most surgeons discuss sport activities after surgery, familiarity with health-enhancing PA recommendations is lacking and the recommendations are only discussed with one-third of patients. Surgeons should pay more attention to the active lifestyle of their patients and can be made more aware of the potential gain in health and fitness for patients after being advised to meet health-enhancing physical activity recommendations. The emphasis should lie on prevention through an active lifestyle, thus preventing chronic diseases and maintain an independent lifestyle while aging. A valuable source of inspiration in this respect is the Exercise is Medicine initiative of the American College of Sports Medicine, which encourages physicians to include physical activity in their treatment plans for patients[31].

## Supporting information

S1 Dataset Dutch health-enhancing physical activity recommendations. (XLSX)

S2 Dataset Overview sports activities age <65 after THA. (XLS)

S3 Dataset Overview sports activities age >65 after THA. (XLS)

S4 Dataset Overview sports activities age <65 after TKA. (XLS)

S5 Dataset Overview sports activities age >65 after TKA. (XLS)
